# Urban Trail Exposure in Winnipeg, Canada: Longitudinal Cohort Development for a 20-Year Difference-in-Differences Analysis of a Natural Experiment with Varied Duration of Exposure Times

**DOI:** 10.23889/ijpds.v9i5.2839

**Published:** 2024-09-18

**Authors:** Heather Prior, Charles Burchill, Jonathan McGavock

**Affiliations:** 1 Manitoba Centre for Health Policy; 2 University of Manitoba; 3 Children’s Hospital Research Institute of Manitoba

## Objective

Develop a cohort to evaluate a real-world experiment of the built environment on the incidence of disease, accounting for movement of people, postal codes, incidence of disease and changing demographics.

## Approach

The City of Winnipeg, Canada built multi-use trails in 2010-2012. We used the administrative data housed at the Manitoba Centre for Health Policy to evaluate the effect of this change in the built environment on the incidence of cardiovascular disease events (CVDE) and risk factors (CVDRF) before (2000-2009) and after (2012-2019) the trails were built. The Manitoba Health Insurance Registry contains postal code and demographic information on nearly all city residents and was utilized for cohort creation and person-time exposure. Individuals’ residential postal codes were linked to geo-spatial data to determine proximity to built trails at 400m, 800m and 1200m. Semi-annual postal code changes accounted for movement within/outside city limits or exclusion from the cohort. Diagnoses from hospital abstract and physician visit data and outpatient prescription dispensations were used to access prevalence and incidence of a CVDE composite measure of congestive heart failure, ischemic heart disease and stroke and a CVDRF composite measure of diabetes, dyslipidemia and hypertension.

## Conclusions and Implications

Leveraging a diverse set of administrative databases, we built a cohort evaluate the effect of building multi-use trails in Winnipeg on the reduction on CVDE and CVDRF. This demonstrates how administrative data can be used to evaluate natural, real-world experiments with minimal direct data measurement or public intrusion, resulting in actionable results to inform public policy.

